# Serological responses to SARS-CoV-2 following non-hospitalised infection: clinical and ethnodemographic features associated with the magnitude of the antibody response

**DOI:** 10.1136/bmjresp-2020-000872

**Published:** 2021-09-23

**Authors:** Adrian M Shields, Sian E Faustini, Marisol Perez-Toledo, Sian Jossi, Joel D Allen, Saly Al-Taei, Claire Backhouse, Lynsey A Dunbar, Daniel Ebanks, Beena Emmanuel, Aduragbemi A Faniyi, Mark Garvey, Annabel Grinbergs, Golaleh McGinnell, Joanne O'Neill, Yasunori Watanabe, Max Crispin, David C Wraith, Adam F Cunningham, Mark T Drayson, Alex G Richter

**Affiliations:** 1 Department of Clinical Immunology Service, University of Birmingham College of Medical and Dental Sciences, Birmingham, UK; 2 Institute of Immunology and Immunotherapy, University of Birmingham, Birmingham, UK; 3 School of Biological Sciences, University of Southampton, Southampton, UK; 4 Birmingham Acute Care Research Group, Institute of Inflammation and Ageing, University of Birmingham, Birmingham, UK; 5 University Hospitals Birmingham NHS Foundation Trust, Birmingham, UK; 6 Institute of Microbiology and Infection, University of Birmingham, Birmingham, UK; 7 Oxford Glycobiology Institute, Department of Biochemistry, University of Oxford, Oxford, UK; 8 MRC Centre for Immune Regulation, University of Birmingham, Birmingham, UK

**Keywords:** respiratory infection, clinical epidemiology, COVID-19

## Abstract

**Objective:**

To determine clinical and ethnodemographic correlates of serological responses against the SARS-CoV-2 spike glycoprotein following mild-to-moderate COVID-19.

**Design:**

A retrospective cohort study of healthcare workers who had self-isolated due to COVID-19.

**Setting:**

University Hospitals Birmingham NHS Foundation Trust, UK (UHBFT).

**Participants:**

956 healthcare workers were recruited by open invitation via UHBFT trust email and social media between 27 April 2020 and the 8 June 2020.

**Intervention:**

Participants volunteered a venous blood sample that was tested for the presence of anti-SARS-CoV-2 spike glycoprotein antibodies. Results were interpreted in the context of the symptoms of their original illness and ethnodemographic variables.

**Results:**

Using an assay that simultaneously measures the combined IgG, IgA and IgM response against the spike glycoprotein (IgGAM), the overall seroprevalence within this cohort was 46.2% (n=442/956). The seroprevalence of immunoglobulin isotypes was 36.3%, 18.7% and 8.1% for IgG, IgA and IgM, respectively. IgGAM identified serological responses in 40.6% (n=52/128) of symptomatic individuals who reported a negative SARS-CoV-2 PCR test. Increasing age, non-white ethnicity and obesity were independently associated with greater IgG antibody response against the spike glycoprotein. Self-reported fever and fatigue were associated with greater IgG and IgA responses against the spike glycoprotein. The combination of fever and/or cough and/or anosmia had a positive predictive value of 92.3% for seropositivity in self-isolating individuals a time when Wuhan strain SARS-CoV-2 was predominant.

**Conclusions and relevance:**

Assays employing combined antibody detection demonstrate enhanced seroepidemiological sensitivity and can detect prior viral exposure even when PCR swabs have been negative. We demonstrate an association between known ethnodemographic risk factors associated with mortality from COVID-19 and the magnitude of serological responses in mild-to-moderate disease.

**Key messages**

- Increasing age, non-white ethnicity and obesity are independently associated with increased IgG antibody responses directed against the SARS-CoV-2 spike glycoprotein.
- This study demonstrates that risk factors associated with mortality from COVID-19 are also associated with increased serological responses in non-hospitalised individuals.
- Anti-SARS-CoV-2 spike glycloprotein antibody response in 46.2% of health care workers who self-isolated during the first wave of the UK COVID-19 pandemic.

## Introduction

In the general population, increasing age, male sex, obesity, non-white ethnicity, socioeconomic deprivation and comorbidities leading to direct or indirect immune suppression are established risk factors associated with mortality from COVID-19.^1^ In hospitalised patients, severe COVID-19 is associated with peripheral blood signatures suggestive of dysregulated interferon responses, T cell exhaustion and high antibody production.^2–5^ Whether high-risk ethnodemographic variables are directly associated with dysregulated immunological responses in severe COVID-19 is not known. Furthermore, whether ethnodemographic variables are associated with differential serological responses against SARS-CoV-2 in mild disease is also unknown.

Healthcare workers provide a unique cohort in which to consider the underlying immunology of SARS-CoV-2 infection. Healthcare workers are at high risk of exposure to SARS-CoV-2 during the course of their work; estimates of infection rates and seroprevalence in cohorts of UK healthcare workers consistently exceed those of the general population.^6–8^ Furthermore, cohorts of healthcare workers tend to be young, ethnically diverse and less comorbid compared with hospitalised patients.

In this study, using a cohort of UK healthcare workers, we define the serological response directed against the SARS-CoV-2 spike glycoprotein of non-hospitalised adults following mild or moderate COVID-19 and explore the relationships between that serological response and ethnodemographic variables that are associated with poor outcome from COVID-19. We also explore associations between disease symptomatology and the serological response. Finally, we consider the cumulative occupational risk faced by UK healthcare workers over the course of the first wave of the COVID-19 and the impact of self-isolation periods on healthcare delivery.

## Methods

A cohort of healthcare workers who had previously self-isolated because they experienced symptoms suggestive of COVID-19 or self-isolated because household contacts had experienced symptoms of COVID-19 were recruited to this study between 27 April 2020 and 8 June 2020. Open invitation to the study was made via UHBFT email to all staff and also advertised via social media. The only predefined exclusion criteria was participation in an existing SARS-CoV-2 vaccine trial or current COVID-19 symptomatology. At the time of this study, SARS-CoV-2 vaccines had not been deployed outside of clinical trials, and antispike antibodies could be used as a surrogate of previous infection. No individuals within this cohort were hospitalised with COVID-19. UHBFT employed approximated 20 000 staff at the time of the study; the cohort represents 4.7% of the entire trust workforce.

All individuals volunteered a venous blood sample that was tested for anti-SARS-CoV-2 spike glycoprotein antibodies using a commercially available IgGAM ELISA that measures the total antibody response (product code: MK654, The Binding Site (TBS), Birmingham). Median time from symptom onset or initial isolation was 45 days (IQR 35.0–54.0 days). There was no significant differences in the median time to sampling by age, ethnicity or weight. The SARS-CoV-2 spike used in the ELISA is a soluble, stabilised, trimeric glycoprotein truncated at the transmembrane region.^9 10^ This assay has been CE marked with 98.3% (95% CI96.4% to 99.4%) specificity and 98.6% sensitivity (95% CI 92.6% to 100%) following PCR proven, non-hospitalised, mild-to-moderate COVID-19. Further serological investigations were undertaken in individuals who were found to be seropositive on this screening assay. TBS anti-SARS-CoV-2 spike plates were also used to assess individual IgG, IgA and IgM antibodies. Serum was prediluted at a 1:40 dilution using a Dynex Revelation automated liquid handler (Dynex, USA). Antibodies were detected using sheep antihuman HRP-conjugated polyclonal antibodies against IgG (1:16 000), IgA (1:2000) and IgM (1:8000) (TBS, UK). Plates were developed after 10 min using TMB core (TBS, UK), and orthophosphoric acid (TBS, UK) used as a stop solution. Optical densities at 450_nm_ (OD_450nm_) were measured using the Dynex Revelation automated liquid handler. IgG, IgA and IgM ratio cutoffs were determined based on running 90 pre-2019 negative serum samples. A minimum specificity of 92% for each ELISA was prespecified, and the OD cut-off for positivity set accordingly using a frequency distribution chart. The IgGAM kit calibrator was then used to establish a cut-off coefficient for each isotype: IgG (1), IgA (0.71) and IgM (0.588). Any ratio values >1 are classed as positive. Any ratio values <1 are classed as negative. The specificity of the individual isotype IgG, IgA and IgM ELISAs were 97.8%, 93.3% and 97.8%, respectively.

At enrolment the following variables were recorded: age, sex, ethnicity, height and weight, number of co-occupants in participants household, whether an individual used public transport in the 2 weeks prior to their isolation period, the dates of their isolation period, their job role, the department in which they worked during the months of March 2020–June 2020, whether they had undergone a previous PCR test for SARS-CoV-2 and the result of that test. Participants were also asked to retrospectively report whether, during their acute illness for which they self-isolated, they suffered any of the following symptoms: cough, shortness of breath, sore throat, fever >37.8°C, fatigue, myalgia, anosmia and diarrhoea. UHBFT inpatient data were sourced by the UHBFT infection control team. The index of multiple deprivation rank from participants home postcodes were sourced from 2019 UK Ministry of Housing, Communities and Local Government statistics^11^ and transformed into a normally distributed score using the function [log(R/(32 844-R)] where R represented the individual rank of a participant’s postcode within the national data.

Data were analysed using Graph Pad Prism V.9.0. Categorical data were compared using the χ^2^ test and optical density distributions using the Kruskal-Wallis test with Dunn’s post-test comparison for individual groups. Seroprevalence data are expressed as a percentage, with binomial confidence intervals calculated using Wilson’s method. The relationship between age, body mass index (BMI), and antibody responses was considered using Pearson’s correlation coefficient. The relationship between antibody levels and time from symptom onset was modelled using a smoothing spline curve with four knots. Multiple logistic regression was performed using seropositivity as the outcome variable. Age, sex, ethnicity, household index of multiple deprivation score, household occupants, whether an individual experienced primary symptoms or isolated due to a household contact becoming unwell and public transport use were included as independent variables. For continuous variables, the OR represents change in odds of seropositivity per unit increase the independent variable. Multiple linear regression was performed using the IgG, IgA and IgM ratios as outcome variables and age, sex, ethnicity, BMI, household index of multiple deprivation score and time from symptom onset as independent variables.

## Results

**Figure 1:**
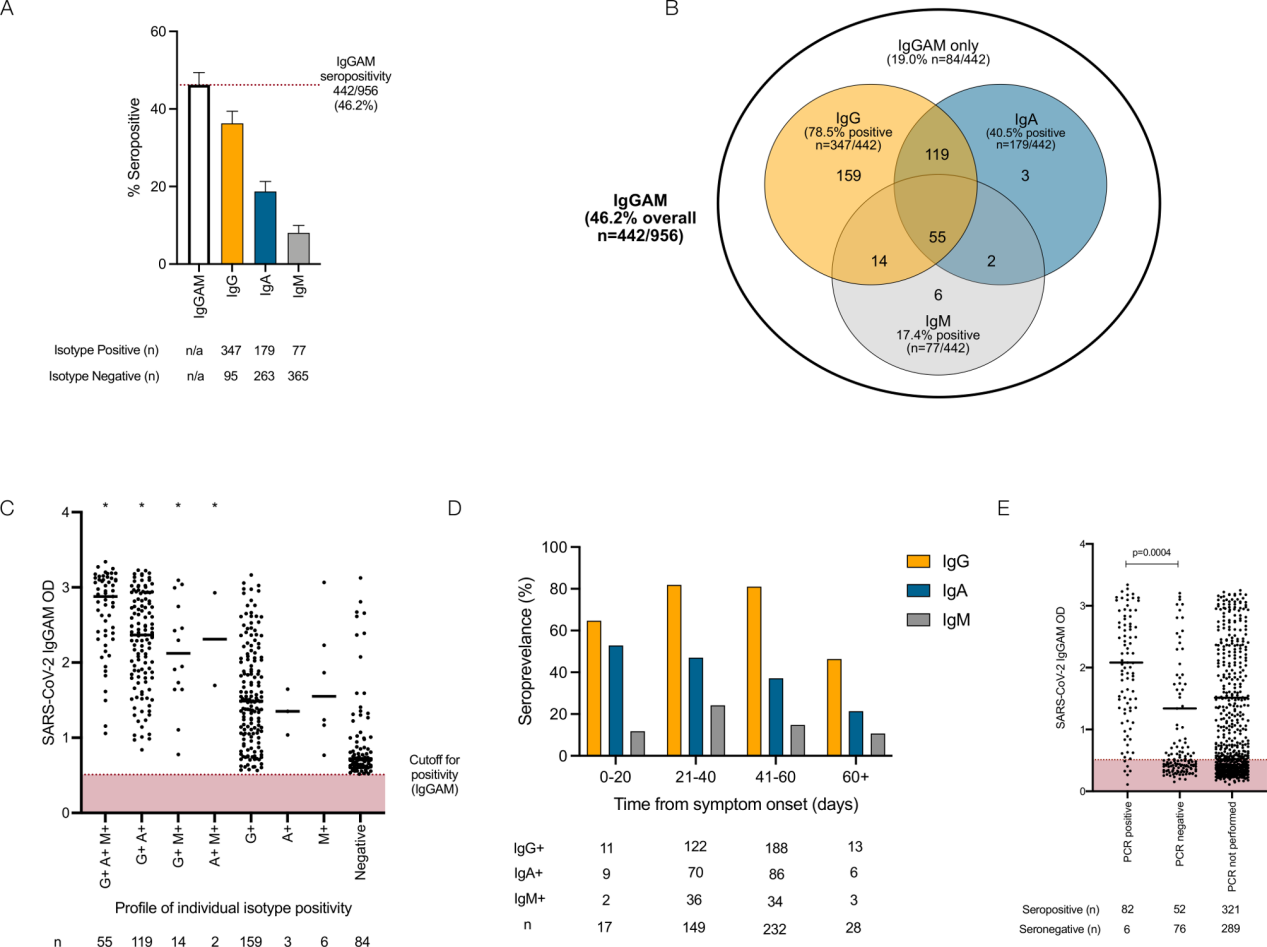
Serological response against the SARS-CoV-2 spike glycoprotein in healthcare workers. (A) IgG, IgA and IgM responses in individuals demonstrating seropositivity in the combined IgGAM ELISA. Error bars represent binomial confidence intervals. (B) Venn diagram illustrating the relationship between IgG, IgA and IgM seropositivity in this cohort. (C) Optical densities (ODs) of the total serum antibody response determined by the combined IgGAM assay, in individuals with different patterns of IgG, IgA and IgM isotype seropositivity. Horizontal bars represent the median of all results above the assay cut-off. *Represents p<0.0001 (Kruskal-Wallis, Dunn’s post-test comparison) of each group compared with the group only detectable using the IgGAM assay. (D) Seroprevalence of IgG, IgA and IgM isotypes in relation to time from symptom onset. (E) Optical densities (ODs) of the total serum antibody response determined by the combined IgGAM assay in symptomatic individuals who had previously undergone PCR testing for the SARS-CoV-2. Horizontal bars represent the median of all results above the assay cut-off.

Nine hundred and fifty-six healthcare workers were enrolled in this study (table 1). Using the combined anti-IgG, IgA and IgM (IgGAM) antibody assay, the overall seroprevalence of anti-SARS-CoV-2 antibodies in the cohort was 46.2% (n=442/956) (figure 1A). Age, sex, number of household co-occupants, public transport use and index of multiple deprivation scores associated with participants home postcodes did not significantly influence seroprevalence (table 1, (online supplemental figure 1). However, ethnicity did have an effect with individuals of black (72.2% seropositive, 95% CI 56.0% to 84.2%) and Asian ethnicity (54.1% seropositive, 95% CI 46.2% to 61.4%) demonstrating the highest seroprevalence (overall χ^2^ 19.2, df 5, p=0.002) (online supplemental figure 1). Individuals who self-isolated because a household contact had experienced symptoms suggestive of SARS-CoV-2 infection (n=162/423) were significantly less likely to be seropositive at the time of the study than those who self-isolated because they experienced symptoms directly (n=243/467) (38.3% vs 52.0%, χ^2^=16.89, z=4.11, df=1, p<0.0001). When these variables were considered in a multiple logistic regression model (table 2), black, Asian and minority ethnic (BAME) ethnicity (OR 1.90 (95% CI 1.30 to 2.81), z=3.26, p=0.001) was the only statistically significant risk factor for seropositivity.

10.1136/bmjresp-2020-000872.supp1 Supplementary data

**Table 1:** Demographics of study population

	All participants, n (%)	Seropositive, n (%)	Seronegative, n (%)	Seroprevalence, (%)	P value
n	956	442	514	46.2	
Age (years)	41.0 (31.0–50.0)	41.0 (32.0–50.0)	40.0 (31.0–50.0)	–	0.69
Sex					
Male	260 (27.2)	110 (24.9)	150 (29.2)	42.6	0.33
Female	679 (71.0)	324 (73.3)	355 (69.1)	47.7	
Not stated	17 (1.8)	8 (1.8)	9 (1.8)	47.0	
Ethnicity					
White	691 (72.3)	294 (66.5)	397 (77.2)	42.5	0.002
Mixed	22 (2.3)	10 (2.3)	12 (2.3)	45.5	
Asian	170 (17.8)	92 (20.8)	78 (15.2)	54.1	
Black	36 (3.8)	26 (5.9)	10 (1.9)	72.2	
Other	25 (2.6)	13 (2.9)	12 (2.3)	52.0	
Not stated	12 (1.3)	7 (1.6)	5 (1.0)	58.3	
Index of multiple deprivation score	780	−0.04 (0.82)	−0.04 (0.77)		0.99

Median and IQRs are provided. Age was compared using a two-tailed unpaired Mann-Whitney test. Categorical data were compared using the χ^2^ test. The index of multiple deprivation scores were compared using an unpaired two-tailed t-test.

**Table 2:** Multiple logistic regression of factors affecting seropositivity

Variable	OR (95% CI)	Z	P value
Age	1.01 (0.99 to 1.02)	0.62	0.53
Sex (female)	1.35 (0.93 to 1.98)	1.56	0.12
Ethnicity (BAME)	1.90 (1.30 to 2.81)	3.26	0.001
Household co-occupants	1.04 (0.91 to 1.20)	0.59	0.55
Index of multiple deprivation score	1.04 (0.84 to 1.28)	0.33	0.74
Primary symptoms	1.22 (0.87 to 1.72)	1.16	0.25
Public transport	0.91 (0.60 to 1.37)	0.46	0.65

Seropositivity at the time of study enrolment was used as the dependent variable. Participants’ age, sex, ethnicity (white vs BAME), number of household co-occupants, the index of multiple deprivation score, whether an individual isolated because they directly experienced symptoms or isolated because a family member experienced symptoms and public transport use in the 2 weeks prior to isolation were used as independent variables. ORs and 95% CIs are provided. The area under the receiver operator curve of this model was 0.58, p=0.0007.

BAME, black, Asian and minority ethnic.

The 442 seropositive individuals had their antibody response characterised further by measuring the individual immunoglobulin isotypes (IgG, IgA and IgM) against the viral spike glycoprotein (figure 1A). IgG antibodies were detectable in 36.3% (n=347/956), IgA antibodies 18.7% (n=179/956) and IgM antibodies 8.1% (n=77/956). The combined IgGAM assay identified 9.9% (n=95/956) of participants who demonstrated a serological response against the viral spike glycoprotein that would not have been detected if IgG detection alone was used in an equivalent assay (figure 1B). The enhanced analytical sensitivity of combined IgGAM detection arises from the identification of seropositivity in individuals who fall below the limit of detection of the equivalent assays that measure individual immunoglobulin isotypes in isolation (figure 1C). Of the 347 individuals who were seropositive for IgG, 50.1% (n=174/347) also demonstrated IgA antibodies in the serum and 19.8% (n=69/347) demonstrated IgM antibodies in the serum (figure 1B). Exclusive IgA or IgM seropositivity was rare (n=3 for IgA, n=6 for IgM). The enhanced sensitivity demonstrated by combined IgGAM detection may facilitate the identification of seropositive individuals beyond 60 days from symptom onset, where detectable IgG seropositivity falls to 46.4% (n=13/28) (figure 1D, online supplemental figure 2). In this study, only 26.6% (n=216/812) of symptomatic participants received confirmatory PCR testing reflecting the lack of access to community testing at the time. The IgGAM assay identified 93.2% (n=82/88) of individuals who had previously tested positive for SARS-CoV-2 by PCR and an additional 52 previously symptomatic individuals who had tested negative by PCR; these individuals had significantly lower antibody levels than those who had tested positive by PCR (figure 1E). Differences in the magnitude of the total antibody response against the spike glycoprotein between PCR positive and PCR negative participants could not be explained by differences in the time allowed for the maturation of the antibody response, which was equivalent between the groups (time from symptom onset: 33.8 days vs 37.1 days, p=0.18).

**Figure 2:**
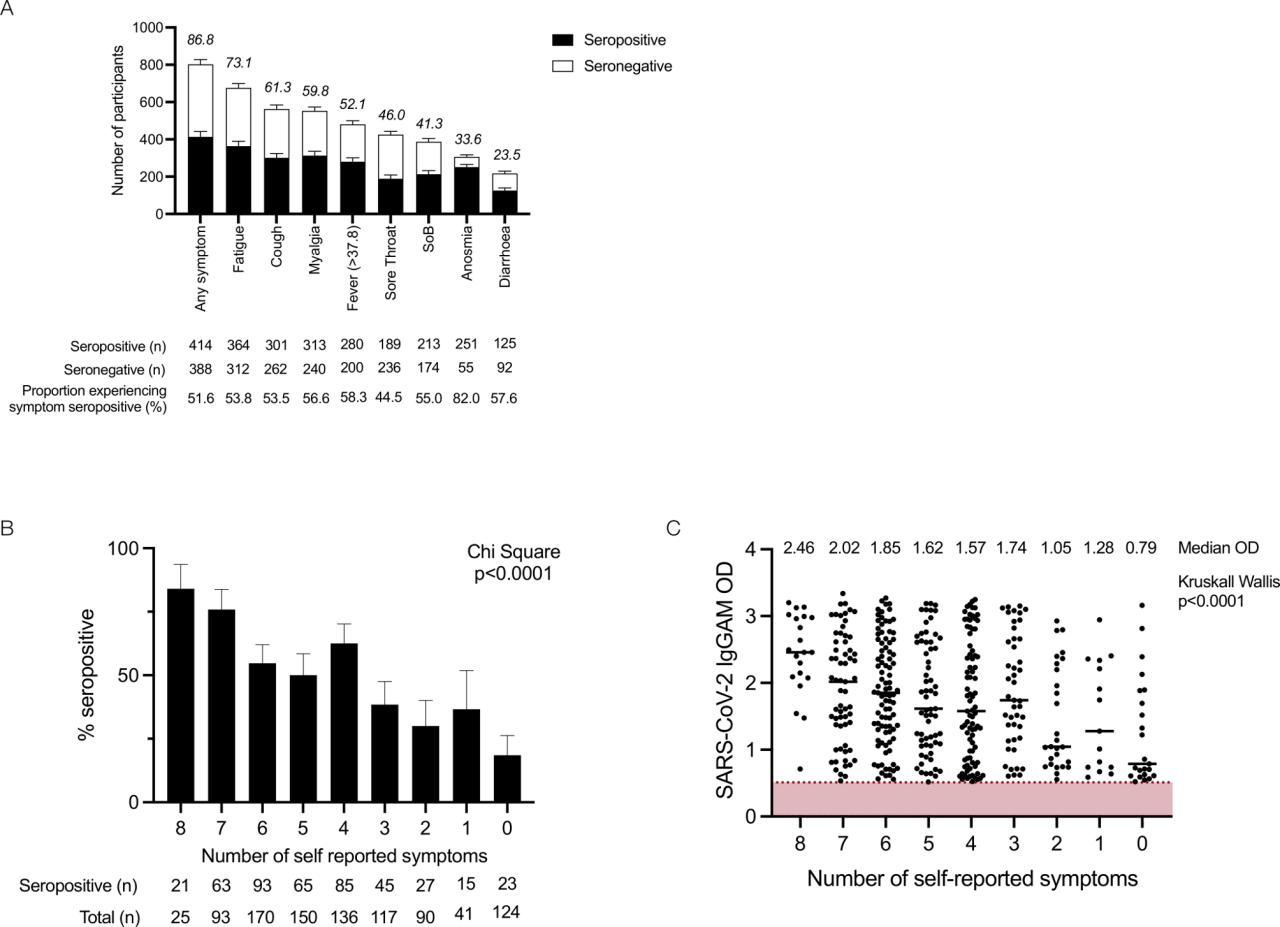
Self-reported symptoms in relation to seropositivity in healthcare workers: (A) self- reported symptoms in relation to seropositivity in healthcare workers. Number bars represent the percentage of participants experiencing symptom. Error bars represent the binomial confidence intervals. (B) Number of self-reported symptoms in relation to seropositivity in healthcare workers; data were compared using χ^2^ (χ^2^=114.8, df=8, p<0.0001). (C) Number of self-reported symptoms in relation to optical density (OD) of the total serum antibody response determined by the combined IgGAM assay.

Participants were asked to self-report symptoms they attributed to SARS-CoV-2 infection at study enrolment (table 3, figure 2A). A percentage of 86.8 (n=812/926) experienced at least one symptom; fatigue, cough and myalgia were the most common symptoms, reported by 73.1%, 61.3% and 59.8% of participants, respectively (table 3). Anosmia was the most sensitive symptom (82.0%) with respect to seropositivity at study enrolment but was reported by only 33.5% (n=306/911) of participants.

**Table 3:** Performance characteristics of self-reported symptoms in relation to seropositivity at study enrolment

Symptom	Number of participants experiencing symptom (n)	Participants experiencing symptoms (%)	Sensitivity (%)	Specificity (%)	Positive predictive value (%)	Negative predictive value (%)
Fatigue	676	73.1	53.8	71.1	83.5	36.2
Cough	563	61.3	53.5	62.9	69.5	46.1
Myalgia	553	59.8	56.6	65.4	71.6	49.4
Fever >37.8°C	480	52.1	58.3	64.7	64.2	58.8
Sore throat	425	46.0	44.5	50.9	43.5	51.8
Shortness of Bbeath	387	41.3	55.0	59.1	48.6	65.1
Anosmia	306	33.6	82.0	70.4	58.4	88.6
Diarrhoea	217	23.5	57.6	55.8	28.6	81.1
Cough or fever or anosmia	752	78.6	54.3	83.3	92.3	33.1

In this cohort of HCW who self-isolated during the first wave of the UK pandemic, the combination of cough and/or fever and/or anosmia was experienced by 78.6% of participants and captured 92.3% of individuals who were seropositive at the time of the study enrolment. The likelihood of an individual testing positive for SARS-CoV-2 antibodies progressively increased with the number of self-reported symptoms (χ^2^ 129.9, df=16, p<0.0001) (figure 2B) as did the magnitude of the antibody response against the spike (figure 2C). Individuals testing positive by PCR reported more symptoms than those testing negative by PCR (average number of symptoms: 5.1 vs 4.6, p=0.02).

The relationship between symptoms and the magnitude of IgG, IgA and IgM responses directed against the SARS-CoV-2 spike glycoprotein was analysed (online supplemental figure 3). Self-reported fever and fatigue were associated with significantly greater IgG and IgA responses against the viral spike glycoprotein, while self-reported diarrhoea was associated with significantly greater IgG responses. These symptoms may be associated with a greater degree of systemic illness arising from SARS-CoV-2 infection.

**Figure 3:**
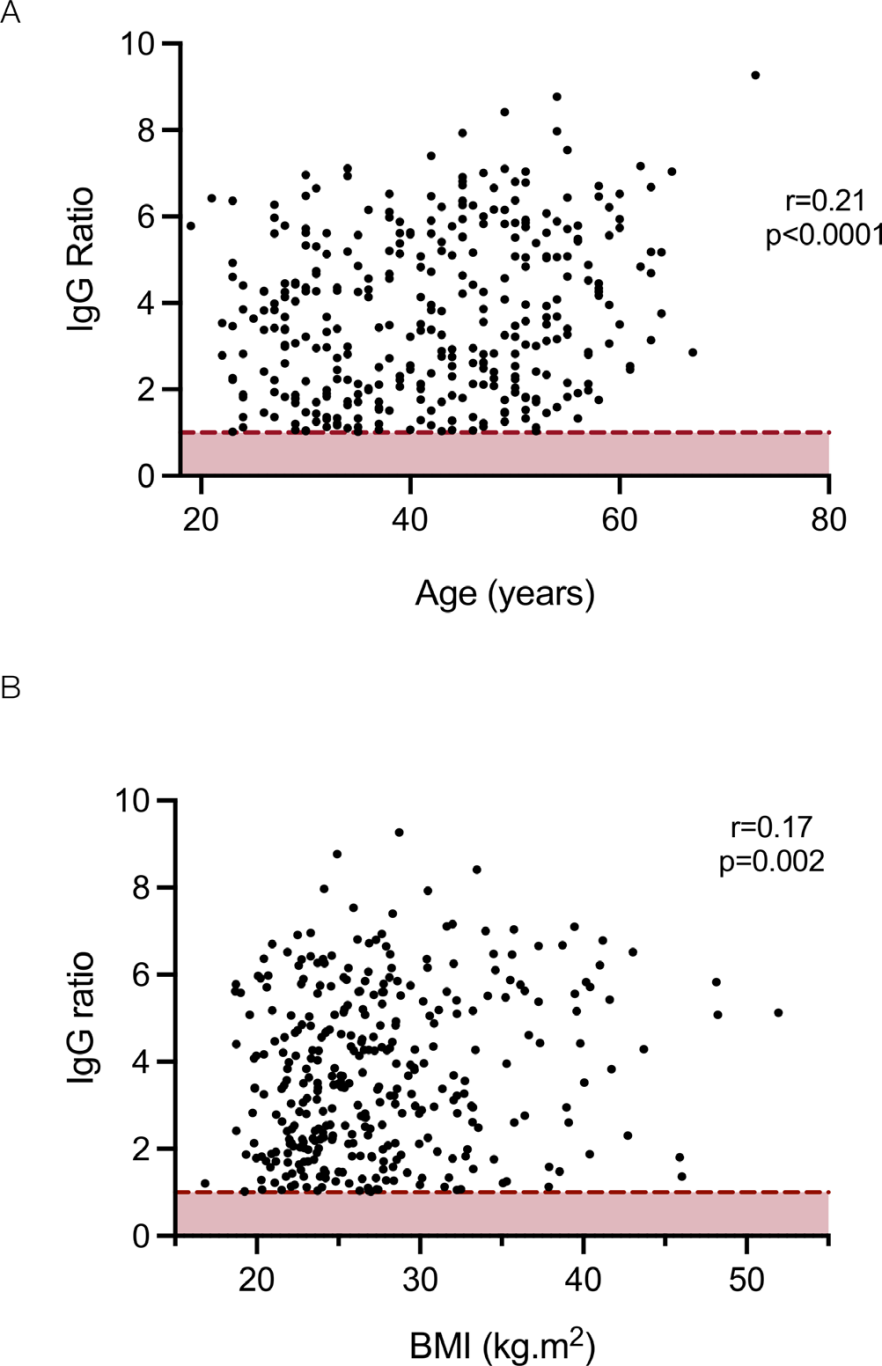
Relationship between age (A) and body mass index (BMI) (B) and the magnitude of the IgG response against the SARS-CoV-2 spike glycoprotein. Dotted red line represents assay cut-off.

The relationship between ethnodemographic variables and the magnitude of IgG, IgA and IgM responses was analysed. Sex did not significantly affect the magnitude of response of any antibody isotype (online supplemental figure 4A). However, increasing age was associated with a higher IgG response against the viral spike glycoprotein (online supplemental figure 4B): a weak but statistically significant positive correlation was observed between age and the magnitude of the IgG response (Pearson correlation r=0.21, p<0.0001) (figure 3A), and when analysed by age brackets, the median IgG response in individuals aged 56–65 years was significantly higher than those aged 26–35 (Kruskal-Wallis statistic 14.0, p=0.02, Dunn’s post-test comparison between 26–35 and 56–65 year old age groups; mean rank difference −65.22, p=0.01) (online supplemental figure 4B). Individuals from all non-white ethnic groups demonstrated higher median antibody levels than white individuals with significantly greater levels observed in Asian individuals compared with white individuals (Kruskal-Wallis statistic 16.9, p=0.005, Dunn’s post-test comparison between white vs Asian ethnic groups; mean rank difference −37.55, p=0.03) (online supplemental figure 4C). Increasing BMI was also associated with increased IgG responses against the viral spike glycoprotein (Kruskal-Wallis statistic 12.1, p=0.03) with a weak but significant correlation (r=0.17, p=0.002) (figure 3B, online supplemental figure 4D). A linear regression model incorporating these variables demonstrated increasing age, non-white ethnicity and increasing BMI were independently associated with greater IgG responses and non-white ethnicity significantly associated with greater IgM responses (table 4).

**Table 4:** Linear regression models of variables affects the magnitude of the antibody response against the SARS-CoV-2 spike glycoprotein

Variable	IgG ratio			IgA ratio			IgM ratio		
	OR (95% CI)	t	P value	OR (95% CI)	t	P value	OR (95% CI)	t	P value
Age	0.03 (0.01 to 0.05)	2.89	0.0043	0.01 (−0.01 to 0.03)	1.21	0.26	0.00 (−0.01 to 0.02)	0.03	0.97
Sex (female)	−0.02 (−0.51 to 0.48)	0.07	0.95	0.17 (−0.27 to 0.60)	0.78	0.45	0.14 (−0.19 to 0.48)	0.84	0.4
Ethnicity (BAME)	0.98 (0.49 to 1.47)	3.95	0.0001	−0.05 (−0.46 to 0.35)	0.25	0.8	0.42 (0.09 to 0.76)	2.53	0.02
BMI	0.07 (0.03 to 0.11)	3.62	0.0004	−0.02 (−0.05 to 0.01)	1.42	0.15	0.00 (−0.03 to 0.04)	0.22	0.83
Time from symptom onset	−0.01 (−0.03 to 0.01)	1.27	0.22	−0.02 (−0.04 to −0.01)	2.76	0.007	−0.01 (−0.02 to 0.01)	1	0.32
Index of multiple deprivation	0.16 (−0.13 to 0.45)	1.09	0.28	−0.10 (−0.36 to 0.16)	0.78	0.44	−0.17 (−0.41 to 0.07)	1.43	0.32
Primary symptoms	0.19 (−0.03 to 0.66)	0.78	0.44	0.27 (−0.14 to 0.67)	1.31	0.19	0.25 (−0.16 to 0.65)	1.24	0.22

The IgG, IgA and IgM ratios were used as dependent variables and participants’ age, sex, ethnicity, body mass index, time from symptom onset, the index of multiple deprivation score, whether an individual isolated because they directly experienced symptoms or isolated because a family member experienced symptoms and public transport use in the 2 weeks prior to isolation were used as independent variables. ORs and 95% CIs are provided. For continuous variables, the OR represents the increase in immunoglobulin ratio associated with each unit increase in that variable. For categorical variables, the OR represents the increase in immunoglobulin ratio associated the variable in parenthesis.

BAME, black, Asian and minority ethnic; BMI, body mass index.

**Figure 4:**
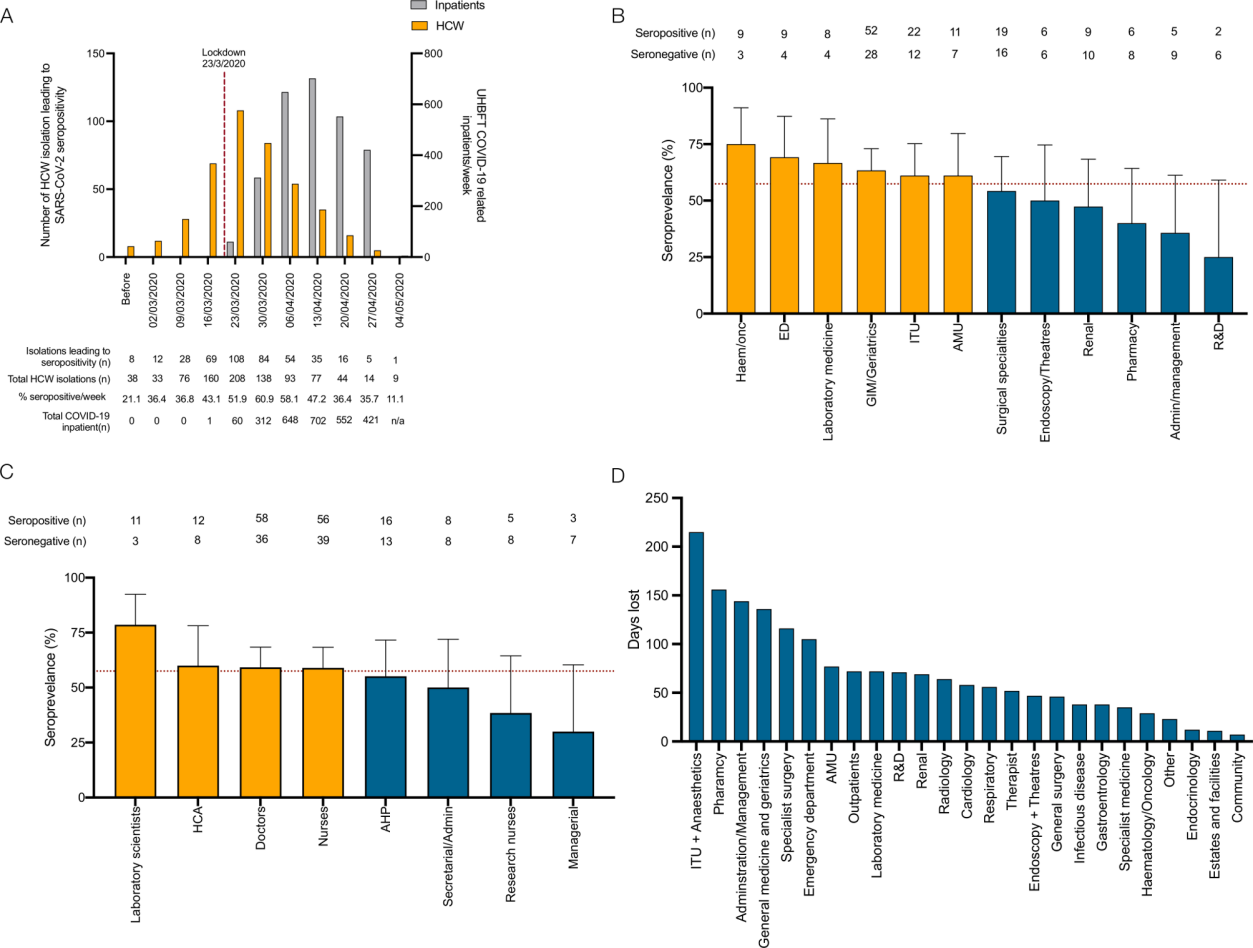
COVID-19 risk in healthcare workers: (A) timing of isolation events in study participants, seroconversion rates (yellow bars) and UHBFT COVID-19 positive inpatients (grey bars) from February to May 2020. (B) Hospital departments and job roles (C) of participants who self-isolated because they directly experienced symptoms following the arrival for the first COVID-19 inpatient at UHBFT; yellow bars represent groups with higher thanaverage seroprevalence, and blue bars represent groups with lower than average seroprevalence. (D) Number of potential days lost due to isolation events in individuals who did not have a PCR test and were found to be seronegative at study enrolment. UHBFT, University Hospitals Birmingham NHS Foundation Trust.

With respect to the timing of infections and occupational risk of exposure in healthcare workers, the proportion of self-isolations associated with seropositivity at the time of study enrolment progressively increased from 21.1% (n=8/38) in February 2020 to a peak of 60.9% (n=84/138) in the week beginning 30 March 2020 before declining during April and May 2020 (figure 4A). By the time of UK national lockdown (23 March 2020), when only 60 proven patients with COVID-19 had been admitted to UHBFT, 53.6% (n=225/420) of self-isolations associated with seropositivity had already occurred. By exclusively considering individuals who had isolated after 23 March 2020, the occupational risk of healthcare workers was reconsidered (figure 4A, B). Seroprevalence in this selected cohort was 57.5% (n=176/306); data were mapped to job roles and hospital departments. Seroprevalence was greater in departments that were directly patient facing (haematology/oncology (75.0%), emergency department (69.2%) and general medicine and geriatrics (63.4%)) and lower in non-patient facing roles (administration/management (35.7%), and research and development (25.0%)). Laboratory scientists had the highest seroprevalence of any healthcare worker group in this study (78.6%); healthcare assistants, doctors, nurses and allied healthcare professionals all had similar seroprevalence (55.2%–60.0%). Multiple logistic regression in this subgroup demonstrated no particular department or job role was at significantly greater risk of seropositivity; however, non-white ethnicity significantly increased the risk of seropositivity in models considering job role and department (online supplemental table 1). Assuming individuals who were seronegative at the time of enrolment in the study were unexposed to the virus, we estimate a total of 1749 working days were lost due to healthcare workers isolating for symptoms that were not attributable to the virus, representing 16.4% of the total working days lost (n=1749/10670). ITU and anaesthetics experienced the greatest burden with a total of 215 working days lost (figure 4D).

## Discussion

Severe COVID-19 is associated with immune dysregulation, multiorgan dysfunction and death. Age, obesity and non-white ethnicity have been independently associated with poor outcome from COVID-19.^1 12^ In this study, we demonstrate that these risk factors are independently associated with greater IgG responses directed against the SARS-CoV-2 spike glycoprotein. Exaggerated serological responses have previously been observed in severe COVID-19^13 14^; however, by conducting this study in individuals with mild disease, it is unlikely our findings are non-specific artefacts of prolonged critical illness. Instead, discreet pathogenic mechanisms are likely to be associated with each variable that require further delineation.

Increasing age is associated with immunosenescence, a phenomenon characterised by complex and progressive immunological changes resulting in increased susceptibility to infectious disease.^15^ As response to vaccination diminishes with age,^16^ it was not anticipated that increasing age would be associated with greater SARS-CoV-2 IgG antibodies. However, this cohort only included individuals of working age and further studies are required to see whether this effect persists in older age groups. Investigation should also consider the quality of the antibody response; for example, increasing age is associated with poor functionality of antipneumococcal antibodies, and discordance has been noted between absolute antibody titres and functionality in the comorbid elderly.^17 18^

Obesity has been postulated to increase mortality from COVID-19 by reducing physiological cardiorespiratory reserve and facilitating a prothrombotic state.^19^ Whether obesity directly affects immunological responses is less clear. Adipose tissue is known to release interleukin-6,^20^ which indirectly induces B lymphocyte antibody production via T lymphocyte derived interleukin-21.^21^ Furthermore, increased BMI is associated with low-grade systemic inflammation evidenced by increased serum C reactive protein, an acute phase protein that is IL-6 dependent.^22 23^ Further studies exploring the relationship between obesity, adiposity, baseline IL-6 levels and the magnitude and quality of antiviral antibody responses may facilitate enhanced patient selection when considering the use of IL-6 blockade in COVID-19 infection.^24^

Non-white ethnicity is associated with poorer outcomes from COVID-19.^1^ It is also associated with either an increased risk of infection from SARS-CoV-2 or an increased proportion of infections that drive serologically detectable antiviral antibody response.^6 8 25^ Socioeconomic differences leading to increased viral exposure have been postulated to account for these differences,^26^ but in this study, household occupancy and deprivation scores associated with a participant’s home postcode were not associated with SARS-CoV-2 seropositivity. BAME ethnicity was, however, independently associated with greater IgG and IgM directed against the SARS-CoV-2 spike glycoprotein. The peripheral immunophenotypes of healthy individuals differs by ethnicity^27^: individuals of African-American ethnicity have significantly greater proportions of type 17 T-follicular helper cells, significantly lower type 1 T follicular helper cells, significantly higher proportions of B cells within their peripheral lymphocyte populations and higher levels of immunoglobulins in comparison with white individuals.^27 28^ Whether an individual’s peripheral immunophenotype correlates with acute antibody responses to SARS-CoV-2 is not known. The epidemiology and genomic architecture underlying differential ethnic susceptibility to antibody-mediated diseases may provide insight into the immune response against COVID-19.^29^ Equally, expression of the ACE-2 receptor, necessary for viral entry, may vary between sex and ethnic groups leading to differential risk of infection on viral exposure.

Our study has implications for future SARS-CoV-2 seroprevalence studies. Previously, we have demonstrated the superior sensitivity of the trimeric, native-like SARS-CoV-2 spike glycoprotein in comparison with the nucleocapsid for the detection of antibody responses in individuals with mild COVID-19.^30^ We now demonstrate that measuring the total antibody response directed against the SARS-CoV-2 spike glycoprotein is more sensitive than measuring an individual immunoglobulin isotype in isolation. It has been postulated that SARS-CoV-2 seroprevalence may be underestimated by not considering systemic IgA responses against virus.^31^ We unequivocally demonstrate that a minority of individuals exclusively mount IgA responses and its independent measurement is unlikely to significantly affect estimates of seroprevalence. However, a combined approach that measures the total antibody responses greatly enhances assay sensitivity in mild disease and should be considered in future seroprevalence studies. Furthermore, these data highlight potential limitations in PCR testing to confirm acute COVID-19. Only 26.6% of symptomatic individuals received a PCR test highlighting the lack of available testing during the first-wave of the COVID-19 pandemic. However, an antibody response was detectable in 40.6% of symptomatic individuals who tested negative by PCR, although the magnitude of this response was significantly less than those who tested PCR positive. This was not explained by differences in the time allowed for maturation of the antibody response, which was equivalent between the groups, but notably, patients who tested PCR negative reported, on average, fewer symptoms than those who tested PCR positive. Previous studies have demonstrated the upper respiratory tract viral load, estimated by PCR cycle threshold values, is equivalent in asymptomatic and symptomatic individuals.^32^ These data would support a hypothesis that some individuals may experience fewer symptoms because they achieve more rapid immunological control over viral replication; this in turn may narrow the window of PCR positivity and highlight potential end-to-end operational insensitivities when PCR is used for the detection of mild disease. Such issues have previously been highlighted in more seriously unwell hospitalised patients^33 34^ and must be very carefully considered when PCR is used as the gold-standard diagnostic reference point to assess the performance of other molecular and serological assays.

With respect to the sustainable delivery of healthcare during future pandemic infections, this study contributes a number of important observations: the overall seroprevalence in this cohort of self-isolating healthcare workers was 46.2%.27.8% of illnesses leading to seroconversion in healthcare workers occurred prior to the arrival of PCR confirmed COVID-19 patients within the hospital environment and 53.6% of illnesses leading to seroconversion had occurred by the end of the following week. Given the median incubation time of the virus is 5 days, these data strongly suggest that the majority of COVID-19 in hospital-based healthcare workers was not acquired from known COVID-19 inpatients in this wave. It also raises the possibility of presymptomatic healthcare workers introducing SARS-CoV-2 into the hospital environment; our previous study demonstrated 2.4% of asymptomatic healthcare workers tested positive for SARS-CoV-2 nucleic acid on nasopharyngeal swabs while at work.^8^

Nevertheless, relatively increased seroprevalence was observed in directly patient-facing workgroups, in comparison with those with minimal or no patient contact suggesting an occupational risk of exposure to SARS-CoV-2 exists and that risk was homogenous for all patient facing groups (55.2%–60.0% seroprevalence in healthcare assistants, doctors, nurses and allied health professional). Of note, laboratory scientists had exceptionally high seroprevalence, possibly due to recirculation of aerosolised virus within temperature-controlled laboratories.^35^ Our data suggest that a considerable number of working days were lost to staff members self-isolating for symptoms that were not molecularly or serologically proven to be COVID-19. As the pandemic has evolved, the symptomatology caused by SARS-CoV-2 infection appears to have changed, particularly in relation to the delta variant.^36^ It is therefore important to continue to monitor the clinical manifestations of COVID-19 so appropriate public health measures can be put in place.

Our study is benefited by the large cohort enrolled but limited by its retrospective nature, its focus on individual of working age and that individuals were asked to self-report symptoms. By selecting self-isolating individuals, the cohort is enriched for individuals who will have had COVID-19: while this allows the study of factors affecting the magnitude of the antibody response, it has the potential to exclude asymptomatic individuals, who demonstrated a 17.1% seroprevalence in our original cross-sectional study of healthcare workers at University Hospitals Birmingham and mounted lower antibody responses overall.^8^ However, asymptomatic individuals were included in this study, accounted for 12.9% of the total cohort, demonstrated an overall seroprevalence of 18.5% and their antibody responses were also lower, on average, than symptomatic individuals.

In conclusion, the variables we identify as affecting the antibody response are known population level risk factors for poor outcome, and it is plausible that an immunological mechanism is implicated in disease pathogenesis. Further studies must continue to explore these associations, particularly in mild disease, to inform COVID-19 pathogenesis.

## Data Availability

Data are available on reasonable request. The anonymised dataset will be made available on reasonable request. Proposals should be directed to the corresponding author.
