# Supplementary material for: Serological responses to SARS-CoV-2 following non-hospitalised infection: clinical and ethnodemographic features associated with the magnitude of the antibody response
Source: BMJ Open Respir Res. 2021 Sep 23;8(1):e000872. doi: 10.1136/bmjresp-2020-000872 (PMC8474079; doi:10.1136/bmjresp-2020-000872)
Supplement: Supplementary data [file bmjresp-2020-000872supp001.pdf]

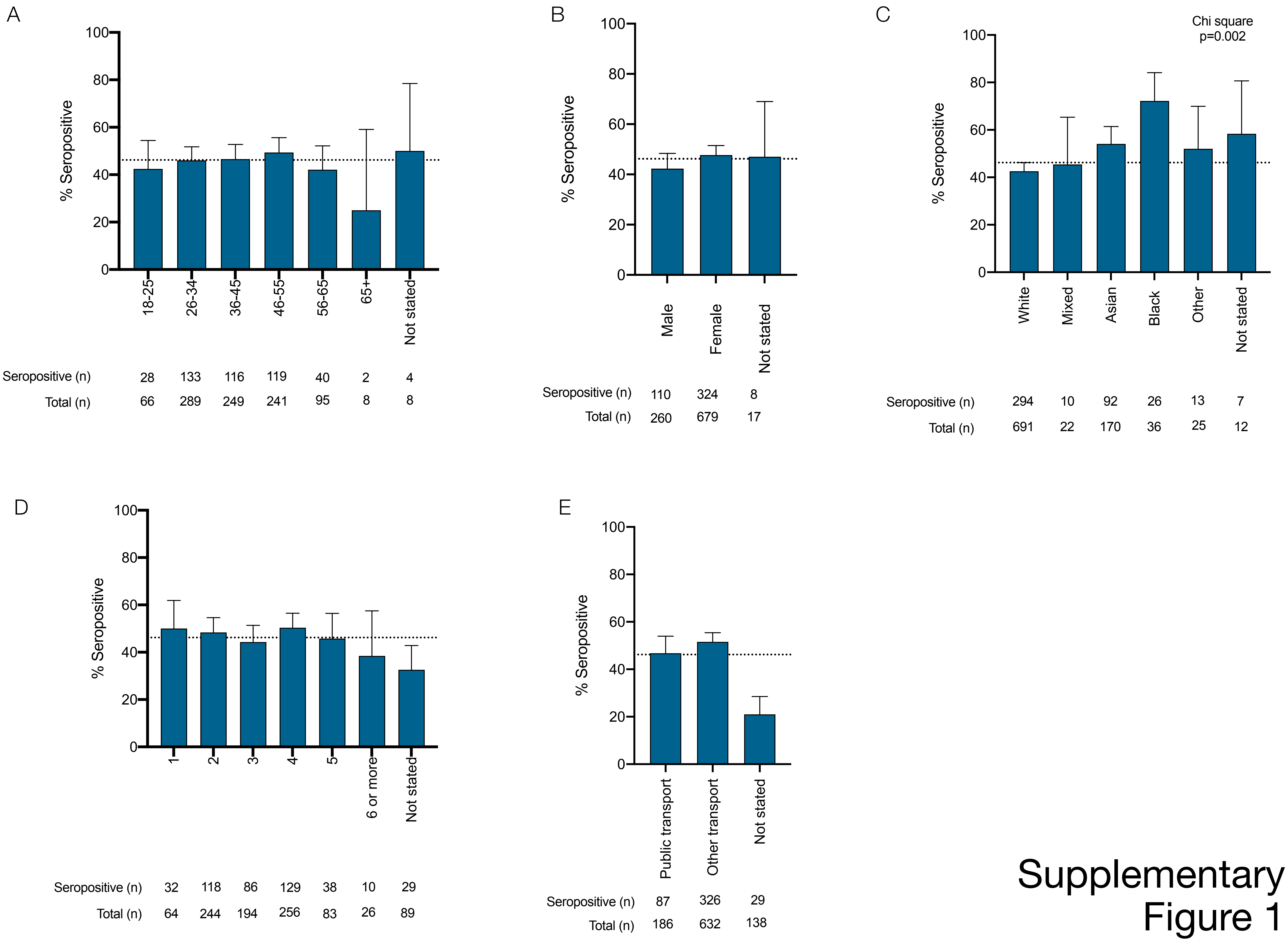

Supplementary Figure 1

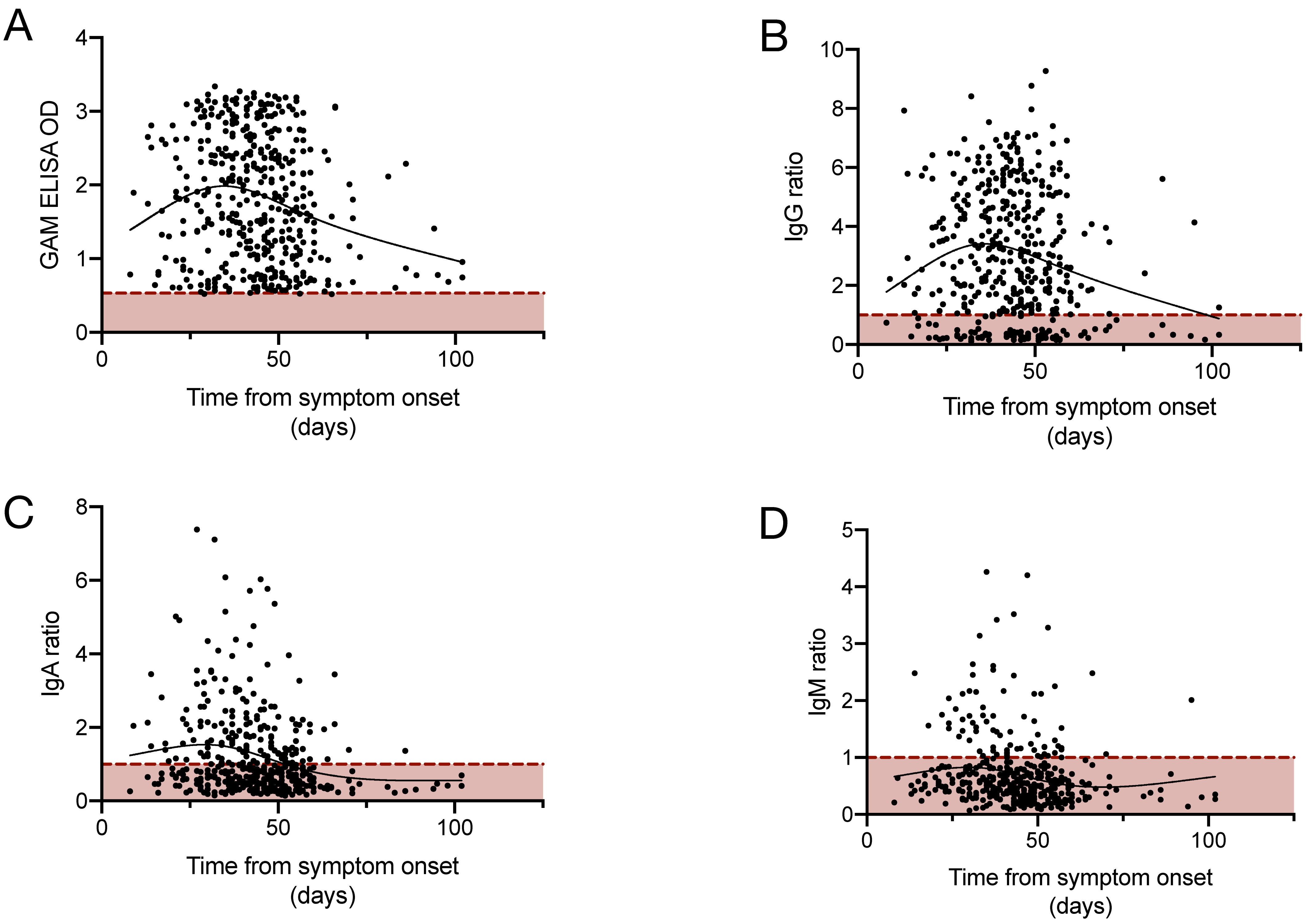

Supplementary Figure 2

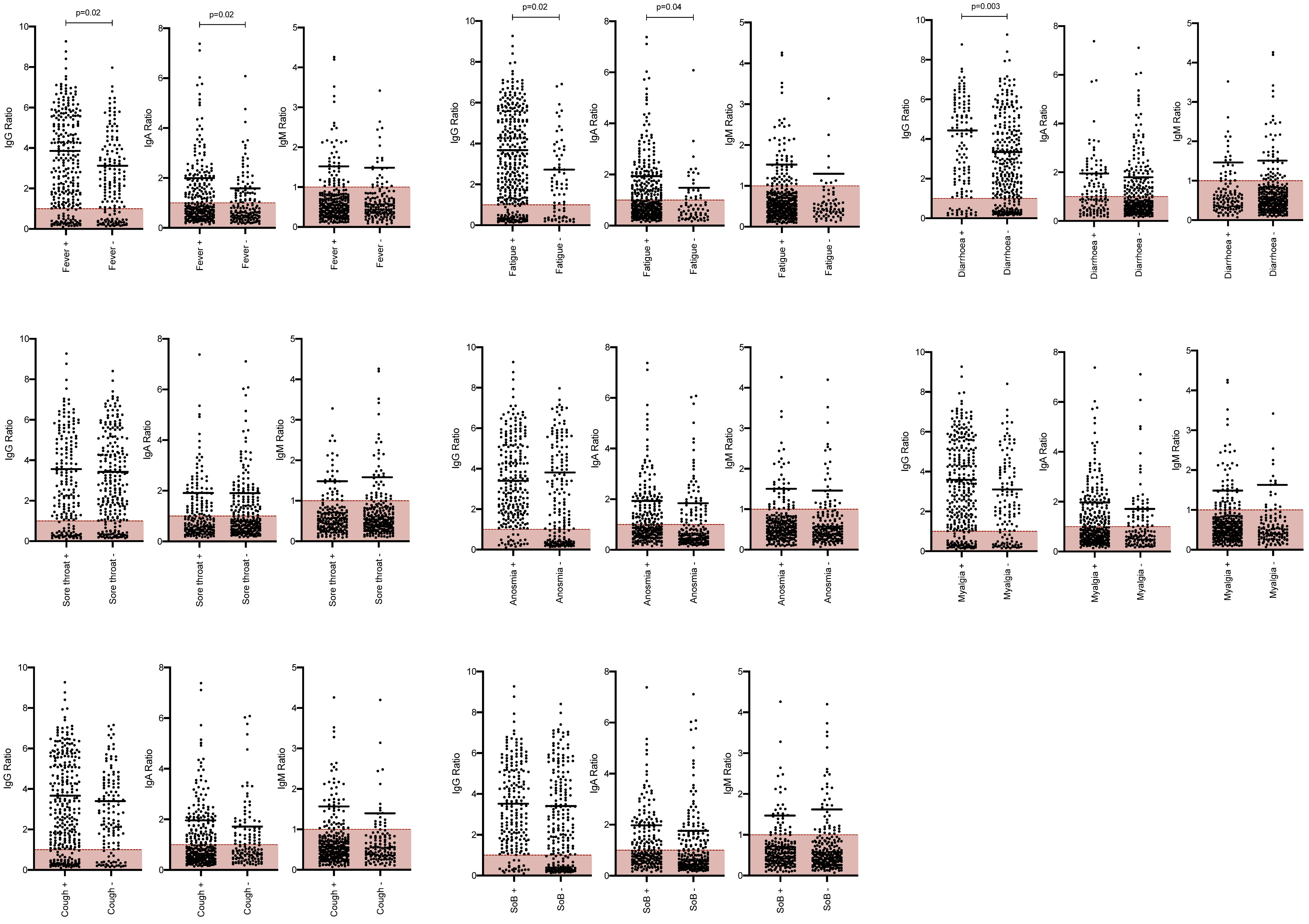

Supplementary Figure 3

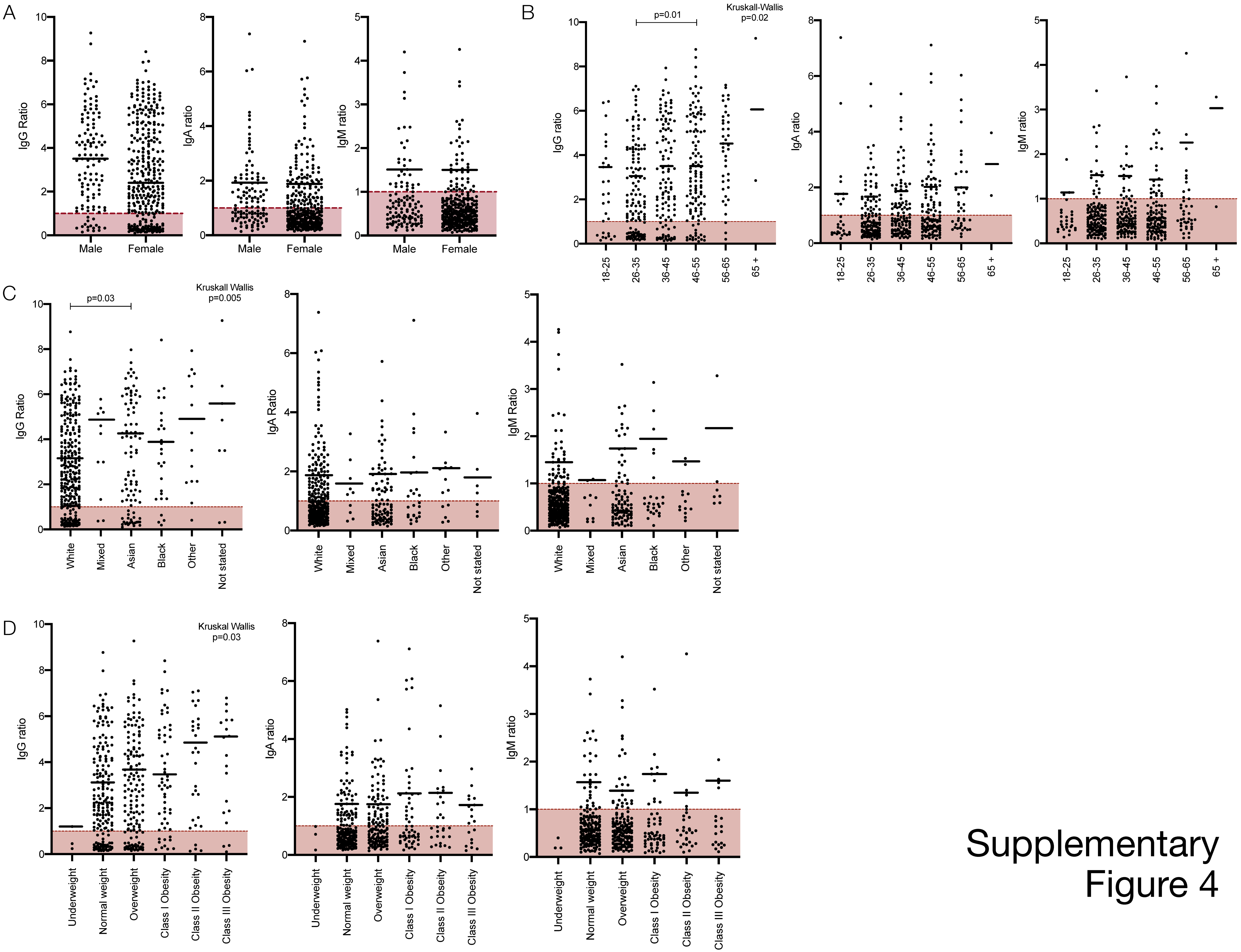

Supplementary Figure 4

Variable	OR (95% CI)	Z	P-value
Age (years)	0.99 (0.97-1.01)	0.91	0.37
Sex (Female)	0.78 (0.42-1.41)	0.83	0.41
**Ethnicity (BAME)**	**1.93 (1.11-3.40)**	**2.31**	**0.02**
Job role : Administration	0.42 (0.07-2.20)	1.00	0.32
Job role : AHP	0.41 (0.07-1.92)	1.09	0.27
Job role : Doctor	0.46 (0.09-1.91)	1.02	0.31
Job role: HCA	0.61 (0.10-3.11)	0.58	0.56
Job role : Lab scientist	1.10 (0.15-7.96)	0.10	0.92
Job role: Managerial	0.19 (0.02-1.22)	1.68	0.09
Job role : Nurse/Midwife	0.61 (0.12-2.43)	0.67	0.50
Job role : Research nurse	0.26 (0.04-1.44)	1.49	0.14

Supplementary Table 1: Multiple logistic regression of seropositivity at time of study enrolment with respect to job role. Area under the curve for this model was 0.61 (CI 0.55-0.68, p=0.0008).

Variable	OR (95% CI)	Z	P-value
Age (years)	0.99 (0.97-1.01)	0.57	0.57
Sex (Female)	0.8 (0.49-1.40)	0.70	0.48
**Ethnicity (BAME)**	**1.93 (1.10-3.43)**	**2.27**	**0.02**
Department: Admin/management	0.54 (0.14-1.97)	0.92	0.36
Department: AMU	1.09 (0.32-3.85)	0.14	0.89
Department: ED	2.05 (0.53-9.05)	1.01	0.31
Department: Endoscopy/Theatres	1.07 (0.26-4.57)	0.09	0.93
Department: GIM/Geriatrics	1.43 (0.59-3.45)	0.80	0.42
Department: Haematology/Oncology	2.67 (0.64-14.05)	1.28	0.20
Department: ITU	1.23 (0.45-3.38)	0.40	0.69
Department: Laboratory medicine	1.35 (0.33-6.16)	0.41	0.68
Department: Pharmacy	0.51 (0.12-1.94)	0.97	0.33
Department: R&D	0.3 (0.04-1.59)	1.32	0.19
Department: Renal	0.67 (0.20-2.16)	0.67	0.50
Department: Surgery	0.98 (0.36-2.67)	0.03	0.97

Supplementary Table 2: Multiple logistic regression of seropositivity at time of study enrolment with respect to department. Area under the curve for this model was 0.64 (CI 0.58-0.71, p<0.0001).
